# An optimized approach for multiplexing single-nuclear ATAC-seq using oligonucleotide-conjugated antibodies

**DOI:** 10.1186/s13072-023-00486-7

**Published:** 2023-04-28

**Authors:** Betelehem Solomon Bera, Taylor V. Thompson, Eric Sosa, Hiroko Nomaru, David Reynolds, Robert A. Dubin, Shahina B. Maqbool, Deyou Zheng, Bernice E. Morrow, John M. Greally, Masako Suzuki

**Affiliations:** 1 Department of Genetics, Albert Einstein College of Medicine, Bronx, NY USA; 2 Departments of Neurology and Neuroscience, Albert Einstein College of Medicine, Bronx, NY USA; 3 Department of Nutrition, Texas A&M University, College Station, TX USA; 4 Present Address: Center for Genetic Medicine, Children’s National Medical Center, Washington, DC USA; 5 Present Address: Thinkcyte Inc., Tokyo, Japan

**Keywords:** Single-cell, Open-chromatin regions, Multiplexing, Assay for transposase-accessible chromatin (ATAC)

## Abstract

**Background:**

Single-cell technologies to analyze transcription and chromatin structure have been widely used in many research areas to reveal the functions and molecular properties of cells at single-cell resolution. Sample multiplexing techniques are valuable when performing single-cell analysis, reducing technical variation and permitting cost efficiencies. Several commercially available methods have been used in many scRNA-seq studies. On the other hand, while several methods have been published, multiplexing techniques for single nuclear assay for transposase-accessible chromatin (snATAC)-seq assays remain under development. We developed a simple nucleus hashing method using oligonucleotide-conjugated antibodies recognizing nuclear pore complex proteins, NuHash, to perform snATAC-seq library preparations by multiplexing.

**Results:**

We performed multiplexing snATAC-seq analyses on a mixture of human and mouse cell samples (two samples, 2-plex, and four samples, 4-plex) using NuHash. The analyses on nuclei with at least 10,000 read counts showed that the demultiplexing accuracy of NuHash was high, and only ten out of 9144 nuclei (2-plex) and 150 of 12,208 nuclei (4-plex) had discordant classifications between NuHash demultiplexing and discrimination using reference genome alignments. The differential open chromatin region (OCR) analysis between female and male samples revealed that male-specific OCRs were enriched in chromosome Y (four out of nine). We also found that five female-specific OCRs (20 OCRs) were on chromosome X. A comparative analysis between snATAC-seq and deeply sequenced bulk ATAC-seq on the same samples revealed that the bulk ATAC-seq signal intensity was positively correlated with the number of cell clusters detected in snATAC-seq. Moreover, when we categorized snATAC-seq peaks based on the number of cell clusters in which the peak was present, we observed different distributions over different genomic features between the groups. This result suggests that the peak intensities of bulk ATAC-seq can be used to identify different types of functional loci.

**Conclusions:**

Our multiplexing method using oligo-conjugated anti-nuclear pore complex proteins, NuHash, permits high-accuracy demultiplexing of samples. The NuHash protocol is straightforward, works on frozen samples, and requires no modifications for snATAC-seq library preparation.

**Supplementary Information:**

The online version contains supplementary material available at 10.1186/s13072-023-00486-7.

## Introduction

Advancing single-cell technologies to analyze chromatin structure and transcription profiles allows us to assess the transcriptional regulatory and transcriptomic landscapes of each cell subtype within a heterogeneous sample. The single-nuclear ATAC-seq (snATAC-seq) assay is based on the assay for transposase-accessible chromatin (ATAC) to define sites of open chromatin in the genome [1, 2], thus identifying regulatory loci at a single-cell resolution. When we assess these regulatory landscapes at single-cell resolution, a single nucleus is captured in an oil droplet containing a barcoded capture bead or sorted into a single well, and then a library is generated for each nucleus in an isolated environment. These steps are usually performed on a sample-by-sample basis, potentially resulting in technical batch effects on the results that are sometimes difficult to resolve computationally at the analysis step. To address this technical difficulty, several multiplexing methods have been developed and widely used to reduce technical batch effects in single-cell RNA-seq (scRNA-seq) studies [3–8]. Recently, Zhang et al*.* reviewed the characteristics of sample-multiplexing approaches used for single-cell sequencing [9]. Among those, the methods using natural genetic variations, such as single nucleotide variants (SNVs), do not require a step prior to generating a scRNA-seq library [6, 10–12]. However, genetic variation-based methods are not applicable to studies lacking genetic differences between samples, such as model organism studies using congenic strains. The most accepted method is cell hashing, defined as pooling sets of cells, utilizing uniquely barcoded oligonucleotide-conjugated antibodies [4] or lipids to tag each of the samples [7]. The first step for both methods is to incubate the cells with an antibody or lipids that bind to an epitope present on the cells being tested in a given sample. Individual samples are labeled with differently barcoded antibodies, and then the samples are combined for a single assay. The conjugated oligonucleotides with a unique barcode will subsequently be sequenced in the single-cell assay, and the unique barcode is utilized to demultiplex the combined samples. This has the effect of minimizing the technical batch effects that would otherwise make assays difficult to compare and reduces the amount of sequencing needed. Barcoded oligonucleotide-conjugated antibodies and lipids are commercially available and widely used in scRNA-seq analysis for demultiplexing. However, since these conjugated oligonucleotides are designed to be captured by oligo-dT scRNA-seq probes, these antibodies or lipids are not applicable to snATAC-seq. Only a few multiplexing techniques are currently available for snATAC-seq [8, 13–15]. These techniques are complicated and sometimes increase the number of steps needed to generate barcoded libraries or require modifying the library preparation method.

In this study, we developed a simple nucleus hashing method, NuHash, to perform snATAC-seq library preparations by multiplexing using hashing oligonucleotides containing a Tn5 tag sequence and a specific barcode that can be sequenced in the single-cell assay. The antibody used in this study has broad reactivities with the nuclear pore complex proteins of vertebrates, Xenopus, and yeast, and our results clearly demonstrated that this new method can improve the multiplexing analysis of human and mouse nuclei from frozen samples with high accuracy. In addition, our analysis comparing snATAC-seq and bulk ATAC-seq peaks suggests that the peak intensities of bulk ATAC-seq can be used to identify different types of open chromatin regions.

## Results

### Design overview

**Fig. 1:**
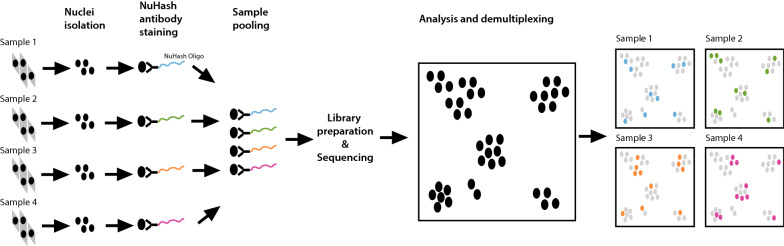
NuHash sample multiplexing schematic overview. Isolated nuclei from each sample are stained with a NuHash oligonucleotide-conjugated antibody containing a unique sample barcode. The stained nuclei from different samples were pooled for snATAC-seq library preparation, followed by massively parallel sequencing. Sequencing reads were then assigned to each nucleus using a nuclei (10x) barcode, and demultiplexing of nuclei from individual samples is carried out using the NuHash reads matching the sample barcode

A schematic overview of sample multiplexing by NuHash is shown in Fig. 1. The detailed NuHash protocol is provided in Additional file 1. Isolated nuclei from cells (fresh or frozen samples) were stained with NuHash oligonucleotide-conjugated antibodies (NuHash antibody) and pooled before loading to the 10 × Genomics system to generate snATAC-seq libraries. Since the NuHash oligonucleotide was conjugated to the anti-Nuclear Pore Complex Proteins antibody and contains adapter sequences required for Illumina sequencing, the NuHash protocol does not require modifications to the 10 × Genomics library preparation or sequencing protocol. After sequencing, the number of NuHash reads per nucleus was counted using a Perl script (Additional file 3.pl). Based on the NuHash read counts, we demultiplexed the nuclei to the sample using a similar method developed for the Cell Hashing technique for scRNA-seq multiplexing [4].

**Fig. 2:**
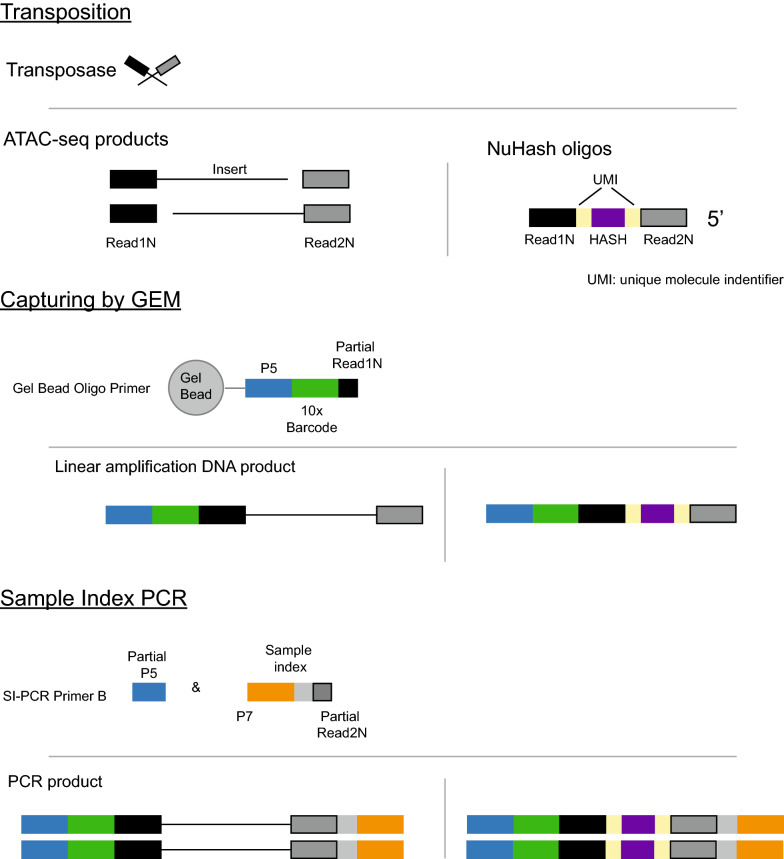
Key steps in NuHash library preparation. The snATAC-seq library preparation and NuHash library preparation are shown in the left and right panels, respectively. The NuHash oligonucleotide is composed of Illumina read1N (black), read2N (gray), two UMIs (yellow), and a sample-specific hash barcode (purple). The read1Ns are used for capturing by GEMs. The captured products are amplified linearly, during which the Illumina P5 adapter (blue) and 10 × barcode (green, a unique identifier for GEM) sequences are also added. Illumina P7 (orange) and sample index barcode (light gray) sequences are added to the products at the PCR amplification step. The full NuHash products contain full-length Illumina sequencing adaptors at both ends, 10 × barcode, two UMIs, NuHash barcode, and sample index barcode (bottom right)

In Fig. 2, we illustrated a flow diagram with the molecular product progression through each step of our library preparation method. The left side shows the snATAC-seq products, and the right side shows the NuHash products. The NuHash oligonucleotide contains two unique molecular identifier sequences (UMI), the sample hashing sequence located between the UMIs, a part of the Illumina Read 1 sequence at the 5′ end, and a part of the Illumina Read 2 sequence at the 3′ end. We included two phosphorothioate bonds at the 3′ end of the sequence to protect the oligonucleotide probes from cell nucleases (Additional file 4: Table S1). The Illumina Read1 sequence binds to the gel beads during the Gel Beads-in-emulsion (GEM) step, and it acquires the complete sequence combination after amplification of the library.

### Optimizing the ratio between hashing antibody and the number of nuclei

The NuHash oligonucleotide sequence contains Illumina P5 and P7 primer sequences, allowing us to amplify the NuHash products with the ATAC-seq products. This is one of the advantages of this method, and it helps to reduce the number of technical steps of library preparation. We tested different ratios of the NuHash antibody to the number of nuclei (Additional file 2: Fig. S1). We isolated nuclei from human CD4 + T cells and stained them with different concentrations of NuHash antibodies. As expected, the higher antibody ratio increased the amounts of NuHash products and reduced the amplification of the ATAC-seq products (Additional file 2: Fig. S1A). We observed that 0.01 µg of NuHash antibody per 50,000 nuclei consistently gave us clear ATAC-seq library characteristic banding patterns [1, 2] after removing large fragments (Additional file 2: Fig. S1B).

### snATAC-seq library preparation

We performed two independent library preparations on mixtures of human and mouse nuclei to assess the accuracy of demultiplexing by alignment results. We used frozen human CD4 + T cells and a mouse hematopoietic progenitor cell line (HPC-7), and each sample was stained with a different NuHash antibody. For the first set, we used two samples: one human CD4 + T sample and one HPC-7 sample. For the second set, we used four samples in total, two samples of CD4 + T cells (male and female, allowing us to assess the accuracy of demultiplexing by alignment results) and two samples of HPC-7. Hereafter, we call the first set 2-plex and the second 4-plex. After staining with different NuHash antibodies, we evenly combined samples of nuclei and adjusted them to 7086 nuclei/sample/µl (2-plex) or 7340 nuclei/sample/µl (4-plex) to target 10,000–20,000 nuclei per sample. We assessed the quality of the libraries before sequencing by examining the fragment analyzer traces and observing the expected banding pattern of the fragment distributions [1, 2] (Additional file 2: Fig. s2).

### Library sequencing and assessing qualities of the NuHash snATAC-seq libraries

We sequenced the libraries on the Illumina NextSeq 500 sequencer (50 bp for read1, 8 bp for i7 index read, 16 bp for i5 index read, and 50 bp for read 2). The paired-end sequence reads were aligned to a human (GRCh38) and mouse (mm10) combined reference genome (refdata-cellranger-atac-GRCh38-and-mm10-2020-A-2.0.0, 10xGenomics) using Cell Ranger ATAC software (version 2.0.0). The total numbers of read pairs and detected cells were 446,720,359 and 16,262 for 2-plex and 353,484,777 and 34,248 for 4-plex, respectively. The alignment statistics are summarized in Additional file 4: Table S2. We assessed the quality of the snATAC-seq libraries using ArchR [16]. From the ArchR analysis, we detected 2266 and 12,880 nuclei aligned to the human genome in 2-plex and 4-plex, respectively. The median fragment numbers per nucleus were 2103 (2-plex) and 1885 (4-plex), and the median transcription start site (TSS) enrichment was 22.69 (2-plex) and 22.808 (4-plex) (Additional file 2: Fig. S3A). The detected duplex was 51 of 2266 (2.3%, 2-plex) and 1658 of 12,880 (12.9%, 4-plex) (Additional file 2: Fig. S3B). In mouse alignment data, we detected 4360 and 13,054 nuclei aligned to the mouse genome in 2-plex and 4-plex, respectively. The median fragment numbers per nuclei were 2731 (2-plex) and 2280 (4-plex), and the median TSS enrichments were 21.09 (2-plex) and 22.69 (4-plex) (Additional file 2: Fig. S3C). The detected duplex was 0 of 4360 (0%, 2-plex) and 1,704 (13.1%, 4-plex) (Additional file 2: Fig. S3D). We observed the expected banding patterns in the insert fragment length distribution in all libraries (Additional file 2: Fig. S3E).

### Accuracy of NuHash

**Fig. 3:**
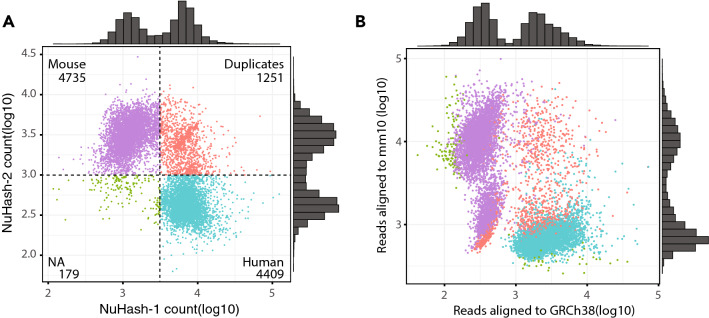
Scatterplot showing the nuclear fragment alignment status of individual nuclei. **A** Based on read counts, nuclei are clustered into quadrants that are human-specific (bottom right, blue), mouse-specific (top left, purple), duplicated (top right, red), or low NuHash counts (bottom left, green). The black dotted lines indicate the cutoff for the counts used for NuHash demultiplexing. **B** Nuclei are colored by the NuHash demultiplexed status according to the ratios of the number of reads aligned to the human or mouse reference genomes

We then selected nuclei with at least 10,000 read counts for further analysis, resulting in 10,574 nuclei (2-plex) and 12,208 nuclei (4-plex). We counted the number of hashing sequences per nucleus. We plotted the number of hash sequence counts for NuHash-1 (human) and NuHash-2 (mouse) using the 2-plex data (Fig. 3A). We observed a clear dissociation between NuHash-1- and NuHash-2-labeled nuclei based on the NuHash count status. In Fig. 3B, we plotted the number of reads aligned to mouse or human reference and colored them based on NuHash demultiplexing status. We assessed nuclear capture by testing the alignment rates of the reference sequence and the counts of NuHash per nucleus. We classified nuclei as singlet if the reads aligned to one of the reference genomes and doublet if the reads aligned to both. For NuHash, we classified nuclei as singlet if the NuHash reads aligned to a single NuHash barcode sequence and duplicate if the NuHash reads aligned to two or more different NuHash barcode sequences. We detected 9144 (86.48%) singlet nuclei and 1,251 (11.83%) duplicate nuclei, while 179 (1.69%) nuclei did not have sufficient NuHash counts (NA). Of the 9,144 singlet nuclei, 4409 (human/NuHash-1) and 4735 (mouse/NuHash-2) were classified based on the NuHash count status (Fig. 3A). We compared the nucleus-assigned classification (human singlet, mouse singlet, and duplicates) based on genome read alignments and NuHash count status. We detected only ten discordantly classified nuclei (8 human genome aligned nuclei with NuHash-2 and 2 mouse genome aligned nuclei with NuHash-1, *p* value < 2.2 × 10^–16^, chi-squared test). The calling accuracy in the 4-plex experiment was comparable to that in the 2-plex experiment (*p* value < 2.2 × 10^–16^, chi-squared test). We detected 12,208 singlets of those 7079 human nuclei with accurate NuHash information, 4979 mouse nuclei with accurate NuHash information, 974 newly classified nuclei, and only 150 nuclei with discordant classifications (Additional file 2: Fig. S4).

### Clustering analysis

**Fig. 4:**
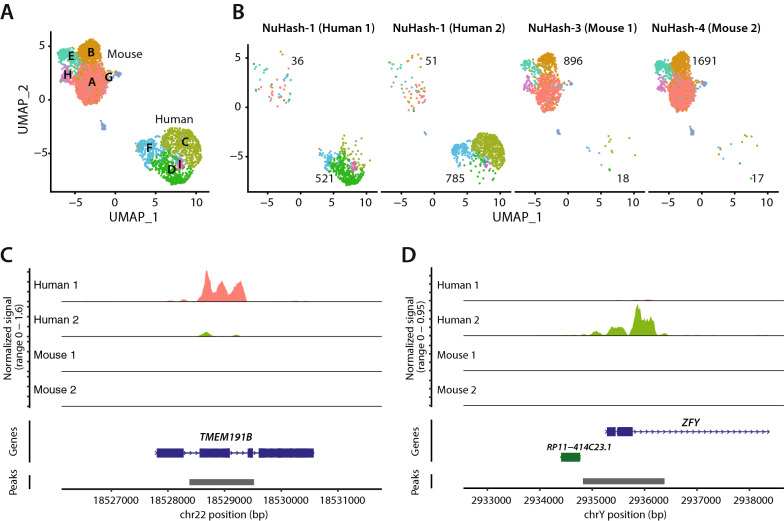
snATAC-seq analysis identified distinct nucleus clusters by open chromatin status. **A** The nuclei were visualized by UMAP and colored by cluster (five mouse and four human clusters). The letters represent the individual samples (or subjects). **B** UMAPs similar to A but plotted separately for each sample. The numbers indicate the detected nuclei in mouse (top) or human clusters (bottom). **C** and **D** Sample-specific read alignment and peak detection at selected genes. **C** In the gene body of TMEM191B, only reads of the human 1 (female) sample were detected. **D** In contrast, only reads of the human 2 (male) sample were detected at the promoter region of the human ZFY gene in chromosome Y

We performed single-cell clustering analysis to identify clusters of cells on human and mouse genome-aligned nuclei in the plex-4 experiment. We aligned the reads to a merged human and mouse reference genome (refdata-cellranger-atac-GRCh38-and-mm10-2020-A-2.0.0, 10xGenomics) to assess the alignment status of each nucleus. We eliminated the nuclei if one of the following conditions was met: (1) the percent of reads in peaks was less than 15% or (2) the number of fragments aligned to peaks was < 2000. A total of 1393 human nuclei and 2622 mouse nuclei passed these thresholds. Among those, we detected 5 clusters in mice (A, B, E, G, and H) and 4 in humans (C, D, F, and I) (Fig. 4A). As expected, a small number of hashed mouse nuclei were clustered with the human nuclei cluster (1.5%) and vice versa (6.3%) (Fig. 4B). To further assess the classification accuracy, we aligned the reads to human (refdata-cellranger-arc-GRCh38-2020-A-2.0.0, 10 × Genomics) or mouse (refdata-cellranger-arc-mm10-2020-A-2.0.0, 10 × Genomics) reads independently. We eliminated the nuclei if one of the following conditions was met: 1) the percent of reads in peaks was less than 15%, 2) the ratio of reads aligned to the genomic blacklist (loci with anomalous, unstructured, or high signal in next-generation sequencing experiments independent of the cell line or experiment [17]) was > 0.05, 3) nucleosome signal was < 0.2 or > 4, and 4) the number of fragments aligned to peaks was < 2000. We analyzed differentially open chromatin regions (OCRs) between Human 1 (female) and Human 2 (male). We identified 29 differential OCRs between male and female human samples (Additional file4: Table S3). The top 4 male-specific OCRs (9 OCRs) were located on chromosome Y. We also found that 5 female-specific OCRs (20 OCRs) were on chromosome X (Additional file4: Table S3). We plotted the top female-specific OCR, which is in the gene body of transmembrane protein 191B (*TMEM191B*) (Fig. 4C), and the top male-specific OCR, which is located in the transcription start site of zinc finger Y-chromosomal protein (*ZFY*) (Fig. 4D), as a representation of sample hashing accuracy. We detected a peak at *TMEM191B* in only Human 1 (female) and at *ZFY* in only Human 2 (male). This result supports the high demultiplexing accuracy of NuHash.

### Bulk ATAC-seq

To assess the accuracy of our NuHash approach, we performed ultradeep bulk ATAC-seq on HPC7 mouse cells to obtain a well-defined open-chromatin region (OCR) profile of the cell line. A total of 8 ATAC-seq libraries from independent cell culture batches were sequenced with the goal of obtaining > 50 million paired-end reads per sample. We excluded one sample that had fewer than 10,000 paired reads. The sequencing statistics of each library are summarized in Additional file 4: Table S4. We obtained a median of 69.8 million paired-end reads per sample (a total of 700.7 million paired-end reads used in the analysis), with a median percentage of reads in OCRs of 58.8%, a median percent of duplication of 0.22%, and detected OCRs ranging from 84,593 to 132,852 (mean = 105,983, standard deviation = 17,024).

### Peak characteristics by the number of cell subtype clusters in which the peak was present

**Fig. 5:**
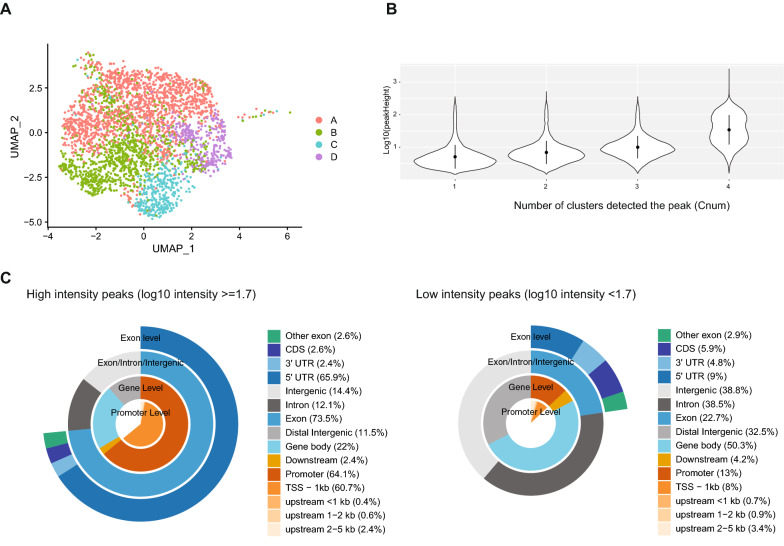
Integration analysis of snATAC-seq and deeply sequenced bulk ATAC-seq highlights a relationship between peak intensity and peak genomic locations. **A** UMAP shows the mouse nuclei identified in the snATAC-seq analysis. **B** Peak intensities of overlapping bulk ATAC-seq peaks were plotted according to the numbers of cell clusters in which the peaks were found in the snATAC-seq analysis. **C** The distribution of peaks over different genomic features detected in all clusters dichotomized by the peak intensities of overlapping bulk ATAC-seq peaks. High-intensity peaks were most prevalent in exonic regions and promoters, while lower-intensity peaks were most common in the gene body and intergenic regions

We assessed the characteristics of snATAC-seq peaks categorized by the number of cell subtype clusters (clusters) in which the peak was present. A higher number of clusters indicates that the peaks were constitutively present, and a lower number means the peaks were cell subtype specific. To increase the robustness of the analysis, we reperformed clustering only for mouse nuclei and selected clusters containing at least 50 nuclei (Fig. 5A). We identified 85,951 peaks and four clusters (A, B, C, and D) in the mouse HPC7 snATAC-seq dataset. Among the identified peaks, 77,987 overlapped with OCRs detected in bulk ATAC-seq (see previous section). Of these 77,987 peaks, 16,810 peaks were detected in only one cluster (Cnum_1), 11,964 in two clusters (Cnum_2), 12,743 in three clusters (Cnum_3), and 36,470 in all four clusters (Cnum_4). When we then looked at the peak intensity of the overlapped bulk ATAC-seq OCRs, they were positively correlated with the number of clusters in which the peak was present (Fig. 5B). The constitutive peaks (Cnum_4) were enriched in promoters and 5’UTR regions (Additional file 2: Fig. S5). Interestingly, the peak heights of the Cnum_4 bulk ATAC-seq OCRs were bimodally distributed, suggesting the existence of stochastic OCRs (low-intensity peaks) and constant OCRs (high-intensity peaks) (Fig. 5B). The high-intensity peaks were enriched in promoters and 5’UTR regions, while the low-intensity peaks were enriched in gene bodies. This finding suggests that the molecular mechanisms that create constitutively open chromatin differ between stochastic and constant OCRs (Fig. 5C).

We also assessed the gene expression and transcriptional regulatory properties of each peak category group. While the GENCODE genes (Mouse Release M15) containing the Cnum_4 high-intensity peak group had higher expression levels than the low-intensity peak group or other Cnum groups containing genes (Additional file 2: Fig. S6A), transcriptionally active putative enhancers with potential bidirectional transcription (TAPEs) [18] (Additional file4: Table S5) evenly overlapped with both the low- and high-intensity peak groups (Additional file 2: Fig. S6B). A comparison of motif enrichment analysis results between low- and high-intensity groups revealed that the CTCF/BORIS motif was significantly enriched in the low-intensity peak group (Additional file 2: Fig. S6C). The proportions of overlap with CTCF ChIP-seq peaks (CTCF peaks) were significantly higher in the Cnum_4 groups (low-intensity, 41.9%, chi-square statistic = 4038.4864, *p* < 0.00001; high-intensity, 40.2%, chi-square statistic = 2105.6447. *p* < 0.00001) than all peaks detected in bulk ATAC-seq (22.14%) (Additional file 2: Fig. S6D). The absolute distances of the CTCF peaks from the TSSs were significantly shorter in the high-intensity peak group than in the low-intensity peak group or all bulk ATAC-seq peaks (Additional file 2: Fig. S6E). Since CTCF and cohesins are master regulators of topologically associating domains (TADs), we also tested whether these CTCF peaks were located in promoter-interacting regions (PIRs) [19, 20]. While only 12.7% of CTCF peaks in low-intensity groups overlapped with PIRs, 60.4% of CTCF peaks in high-intensity groups overlapped with PIRs, suggesting that functional CTCF peaks might be enriched in high-intensity groups.

## Discussion

In this study, we developed a simple nucleus hashing method, NuHash, to perform multiplexing using a high-throughput droplet-based snATAC-seq platform (e.g., 10X Genomics) that is based upon a similar principle to the Cell Hashing technique for scRNA-seq multiplexing [4]. We designed NuHash oligonucleotides containing partial adaptor sequences of Illumina sequencing, UMIs, and barcode sequences unique to each antibody. NuHash oligonucleotides were conjugated to anti-nuclear pore complex proteins. Therefore, no modifications of the 10 × Genomics method are needed. To assess the capabilities of NuHash, we tested the assay of two (2-plex) or four (4-plex) human and mouse pooled samples. We detected 9144 and 12,208 tagged single nuclei, respectively. Both 2-plex and 4-plex analyses showed high accuracy of demultiplexing samples using the barcode sequence of the conjugated oligonucleotide; only 0.11% and 1.23% of nuclei showed mismatched calling between hashing results and aligned DNA results, respectively. In addition, we detected sample-specific peaks between the male and female human samples. Four out of nine male-specific OCRs were located on chromosome Y, and five out of 20 female-specific OCRs were located on chromosome X. We observed sample-specific enrichment in those regions. These results indicated that our simple nucleus hashing approach, NuHash, can effectively demultiplex the combined samples in the snATAC-seq analysis.

To date, several multiplexing techniques have been described to perform high-throughput droplet-based snATAC-seq, such as dsciATAC‑seq [14], CASB [8], and SNuBar [15]. The dsciATAC‑seq method uses indexed Tn5 transposon complexes to tag the samples [14]; CASB tags the samples using concanavalin A with biotinylated oligonucleotides and streptavidin [8]; and SnuBar adds barcoded oligonucleotides at the tagmentation step before partitioning on a microfluidic chip [15]. Both dsciATAC‑seq and SnuBar tag the samples at the Tn5 tagmentation step, and these techniques require modifications of the snATAC-seq library preparations. Moreover, dsciATAC‑seq requires a customized Tn5 enzyme, which creates technical challenges and additional costs. While CASB tags the samples before the tagmentation step and does not require modification of the snATAC-seq library preparation, it uses a secondary binding strategy, which might also cause technical difficulties, to generate the tag information. Our NuHash method uses oligonucleotide-conjugated antibodies and contains the necessary adaptor sequences for generating snATAC-seq libraries; therefore, it is more straightforward and simpler and does not require any complicated optimization for tagging or modifying the library preparation procedure. The antibody we used here has reactivities in a broad range of species; thus, the NuHash system has broad usage as it can be applied to different species, such as humans, mice, yeast, and nematodes. Moreover, our NuHash method employs a stable oligonucleotide-conjugated antibody for indexing, allowing it to operate on frozen samples. Using cell-specific or protein modification-specific antibodies for conjugation, this technology can investigate specific cell populations, including rare ones, across a multitude of samples. This presents a notable advantage. Therefore, we firmly believe that NuHash technology is the most straightforward and stable method currently published, with many possible applications.

Comparisons between snATAC-seq and deeply sequenced bulk ATAC-seq of mouse hematopoietic progenitor cells revealed the importance of studying peak intensities of bulk ATAC-seq data in analyses. Our results indicated that the bulk ATAC-seq peak intensities were positively correlated with the number of cell clusters detected, i.e., lower intensity peaks were called in a subset of cell clusters. However, some low-intensity peaks were detected in all cell subtypes (Cnum_4 peaks), resulting in a bimodal distribution of bulk ATAC-seq peak intensity in the Cnum_4 peaks. This result suggests the existence of both metastable and stochastic OCRs in the mouse genome. In this study, we found that these metastable and stochastic OCRs have different expression regulatory characteristics, suggesting that we need to interpret high-intensity and low-intensity peaks separately when interpreting data from bulk ATAC-seq.

While the current snATAC-seq technique is designed to generate a library from up to 10,000 nuclei, we speculate that the analyzable nucleus number of snATAC-seq will be increased by advancing technology in the near future, given how the latest scRNA-seq method can analyze up to 3,500,000 nuclei with multiplexing. We believe snATAC-seq with multiplexing should become a common technique in the future. Notably, the antibody used in this study has reactivities with nuclear pore complex proteins of vertebrates, *Xenopus*, and yeast; therefore, this hashing method could be used in assays for other species, as well as in multiomics studies that use isolated nuclei. There are some limitations of our NuHash method: for instance, performing multiplexing analysis requires another 10 to 15% of sequence reads for demultiplexing. Additionally, titrating antibodies using the same sample type (species, tissue, and cell types) might be required before running a large set of samples to obtain high-quality libraries. However, these limitations are not unique to NuHash, as most other multiplexing techniques have similar limitations.

## Conclusion

We have developed a new simple method, NuHash, to perform snATAC-seq analysis with multiplexing using oligo-conjugated anti-nuclear pore complex proteins, which can be used for frozen samples, and demonstrated the accuracy of demultiplexing of NuHash. An integration analysis of snATAC-seq with NuHash and deeply sequenced bulk ATAC-seq datasets revealed the importance of considering peak intensity in interpreting the bulk ATAC-seq results.

## Materials and methods

The detailed NuHash protocol is provided in the Additional information document.

### Custom oligonucleotide-conjugated nucleus hashing antibody

The sequences of the custom oligonucleotides are listed in Additional file4: Table S1. The custom oligos were synthesized by Integrated DNA Technologies and conjugated to an anti-nuclear pore complex protein antibody (Clone Mab414, BioLegend) by BioLegend. The oligonucleotide**-**conjugated antibodies were aliquoted and stored at 4 °C until use.

### CD4 T-cell isolation from whole blood

CD4 T cells were isolated from 10 to 20 ml of peripheral blood using an EasySep Direct Human CD4 + T-cell Isolation kit (Stem Cell Technologies, cat #19662). The isolated CD4 T cells were stored in Crystor CS10 (Stem Cell Technologies, cat #07930) at − 80 °C until use (50,000 cells per tube). This study was approved by the Albert Einstein College of Medicine Institutional Review Board (IRB Protocol# 2021-12969 and 2007-272).

### HPC-7 Hematopoietic progenitor cell

The hematopoietic progenitor cell line HPC-7 was kindly gifted by Dr. Britta Will at Albert Einstein College of Medicine. HPC-7 cells were maintained at a density of 2–10 × 10^5^/ml in Iscove’s modified Dulbecco’s medium (Invitrogen) supplemented with 50 ng/ml mouse stem cell factor (Gemini Bio-Products), 1 mM sodium pyruvate (Invitrogen), 6.9 ng/mL α-monothioglycerol (Sigma-Aldrich), 5% bovine calf serum and penicillin‒streptomycin (Invitrogen).

### Nucleus isolation

Frozen human CD4 T cells were thawed with a series of dilutions with prewarmed 10% FBS (Gemini Bio, Cat# 100-106)-supplemented RPMI-1640 medium (Gibco, cat# 11875093) and washed with 0.04% BSA (Sigma-Aldrich, cat# 126609-5GM) in PBS(−) (Gibco, cat# 10010031). Freshly collected HPC-7 cells were washed with 0.04% BSA in PBS(−) before lysis. We lysed pelleted cells with lysis buffer (10 mM Tris–HCl (pH 7.4), 10 mM NaCl, 3 mM MgCl_2_, 0.1% Tween-20, 0.1% NP-40, 0.01% digitonin (Invitrogen, cat# BN2006)) for 3 min on ice, then we washed nuclei with wash buffer (10 mM Tris–HCl (pH 7.4), 10 mM NaCl, 3 mM MgCl_2_, 1% BSA, 0.1% Tween-20) and followed by washing with staining buffer (2% BSA, 0.01% Tween-20 in PBS(−)). The isolated nuclei were resuspended in 10 µl of staining buffer. The number of nuclei in the solution was counted using a hemocytometer (Fisher Scientific, cat# 0267151B).

### NuHash antibody staining

We stained isolated nuclei with a NuHash antibody in the staining buffer. After counting the number of nuclei per µl of nuclei suspension, we adjusted the number of nuclei to 150,000–500,000 in 100 µl of the staining buffer. After Fc blocking with 10 µl of FcX (BioLegend, cat# 422301/101319) for 10 min on ice, we stained nuclei with NuHash antibody for 20 min on ice and then washed the nuclei three times with 1 ml of staining buffer. After the last wash step, we removed all supernatant and resuspended the nuclei in 5 µl of Diluted Nuclei buffer (10 × Genomics, PN-20000153/20000207). The number of nuclei in the solution was counted using a hemocytometer (Fisher Scientific, cat# 0267151B).

### Chromatin accessibility assay

Chromatin accessibility (ATAC-seq) assays were performed according to the Omni-ATAC protocol with some modifications [2]. Freshly isolated nuclei were spun down (500 RCF, 10 min, 4 °C), the supernatant was carefully removed, and the nuclei pellet was resuspended in 50 µL of the transposase reaction mix including 25 µl of 2 × TD buffer (Illumina, cat# 15027866), 2.5 µl of transposase (Illumina, cat# 15027865), 16.5 µl of PBS(−), 0.5 µl of 1% digitonin (Promega, cat# G9441), 0.5 µl of 10% Tween-20, and 5 µl of nuclease-free H_2_O. The transposition reaction was performed at 37 °C for 30 min, followed by purification using a Zymo DNA Clean and Concentrator-5 kit (Zymo Research, cat# D4013). Purified, transposed DNA was eluted in 11 µL of EB elution buffer and stored at − 20 °C until amplification. For indexing and amplification of transposed DNA, we combined the following for each sample: 10 µL of transposed DNA, 25 μl of NEBNext High-Fidelity 2 × PCR Master Mix (New England Biolabs, M0541S), 2.5 µL each of Nextera i5 and i7 indexed amplification primers (Nextera Index Kit, Illumina, FC-121-1011) and 10 μl of nuclease-free H_2_O. The PCR was carried out using the following conditions: one cycle of 72 °C for 5 min and 98 °C for 30 s; ten cycles of 98 °C for 10 s, 63 °C for 30 s and 72 °C for 1 min; and a hold step at 4 °C. The libraries were purified with double-sided bead purification using AMPure XP (Beckman Coulter, catalog # A63880) and eluted in 20 µL of elution buffer. The library quality was assessed by Bioanalyzer High-Sensitivity DNA Assay. The ATAC-seq libraries were quantified by a Qubit HS DNA kit (Life Technologies, Q32851). 150 bp, paired-end sequencing was performed on a HiSeq 2500 Illumina instrument at Novogene Co., Ltd.

### Antibody titration analysis

We performed antibody titration assays to obtain optimal concentrations for the NuHash antibody. We stained isolated 50,000 human CD4 T-cell nuclei at ratios of 0.01 µg of NuHash antibody per 10,000, 25,000, and 50,000 nuclei. After staining, the nuclei were washed three times with the staining buffer, and libraries were generated using the Omni-ATAC protocol [2]. The ratios of NuHash products and the ATAC-seq products were assessed by Bioanalyzer High-Sensitivity DNA Assay.

### Single-nuclei ATAC-seq library preparation

Single-nuclei ATAC-seq libraries were generated using a Chromium Single Cell ATAC seq library preparation kit (10 × Genomics, cat# PN-1000111/PN-1000084). The nuclei stained with nucleus-hashing antibodies were adjusted at a concentration of 7000 nuclei/µl or 7700 nuclei/µl, and two or four samples stained with different antibodies were combined into a tube to run the library preparation by following the manufacturer’s instructions. The library preparation step was performed at the Genomics Core at Albert Einstein College of Medicine. After amplification, we sequenced the libraries as follows: 50 bp for read1, 8 bp for i7 index read, 16 bp for i5 index read, and 50 bp for read 2. Sequencing was performed at the Epigenomic Shared Facility at Albert Einstein College of Medicine.

### NuHash analysis

The sequence reads were aligned to a reference genome that combined the human (GRCh38) and mouse (mm10) reference genomes (refdata-cellranger-atac-GRCh38-and-mm10-1.2.0.tar.gz, 10 × Genomics) using Cell Ranger ATAC ver 1.2.0 (10 × Genomics). The numbers of NuHash sequences mapped to each of the valid cells from Cell Ranger were counted using a Perl script, which is available as supplemental material.

### snATAC-seq analysis

We realigned the obtained sequences to human GRCh38 (refdata-cellranger-atac-GRCh38-1.2.0, 10 × Genomics) or mouse mm10 (refdata-cellranger-atac-mm10-1.2.0, 10 × Genomics) references using Cell Ranger ATAC ver 1.2.0 (10 × Genomics), separately. The quality of the libraries was assessed using ArchR [16]. The obtained peak counts were analyzed using Signac [21] and Seurat [22].

### Bulk ATAC-seq analysis

The bulk ATAC-seq libraries were analyzed, as we previously reported [23]. After assessing the qualities of the sequences using FastQC [24], the adapter sequences were trimmed with Cutadapt [25]. The adapter and quality-trimmed sequences were aligned to the mouse mm10 reference using BWA-mem software [26]. The peak-calling analysis on aligned reads was performed using MACS2 [27]. We calculated the reads in peak (RiP) with the ChIPQC Bioconductor package [28]. We used ChIP-R to identify reproducible peaks [29].

### Identification of transcriptionally active putative enhancers

We combined all bulk ATAC-seq library-aligned reads and performed peak-calling to generate a master OCR list for the identification of enhancer regions and Transcriptionally Active Putative Enhancers (TAPE), as previously reported [18]. We downloaded and used the three HPC7 cell RNA-seq datasets from the NCBI GEO website (GSE132724) [30]. The ATAC-seq results were merged and recentered using BEDTools (version 2.28). All peaks smaller than 146 bp were removed to create a list of regions of open chromatin. Seqmonk (Babraham Institute) was used to identify intergenic regions of open chromatin (iROCs) and high-quality TAPEs by filtering probe lists against known UCSC, Ensemble, and RefSeq gene curated lists for mm10. A final list of 145 high-quality bidirectional TAPEs was identified. To map TAPEs to associated genes, transcription start sites located within 1 Mb upstream or downstream from the center of the TAPE were identified. Then, Pearson’s correlations were calculated using counts for the TAPEs and associated genes.

### Assessing OCR characteristics using publicly available datasets

To assess the characteristics of the identified OCRs, we downloaded and used publicly available HPC7 datasets: a series of hematopoietic transcription factor ChIP-seq (GSE22178) [31] and CTCF ChIP-seq and promoter capture Hi-C (pCHiC) (GSE129478) [32]. The overlap status was assessed using the findOverlapsOfPeaks function of the ChiPpeakAnno Bioconductor package [33]. OCR annotation was performed using the annotatePeak function of the ChIPseeker Bioconductor package [34] with the TxDb.Mmusculus.UCSC.mm10.knownGene annotation database [35]. Transcription factor binding motif enrichment analyses were performed using findMotifsGenome.pl from HOMER with the mm10 reference [36]. All publicly available datasets were lifted to the mm10 reference if the original analysis was performed on a different reference version, and MAC2 was used to call peaks on the bedGraph files.

## Supplementary Information

**Additional file 1**. Detailed method for NuHash library preparation.

**Additional file 2**: **Figure S1**. Optimizing NuHash antibody concentration to the number of nuclei. We stained the human CD4 + T-cell nuclei with different concentrations of NuHash antibody and generated bulk ATAC-seqlibraries to assess the proportions of NuHash and ATAC-seq products.We stained with an antibodyat three different concentrations. The panels show the fragment distributions of each library before removing large fragments by size selection.We stained with two different antibodiesat a 0.01 µg/50 k nuclei concentration. The panel shows the fragment distribution after removing large fragments by size selection. **Figure S2**. Library fragment distributions of NuHash scATAC-seq. NuHash antibodies were used to generate scATAC-seq libraries by multiplexing twoand four samples. The panels show the fragment length distributions of the scATAC-seq library final products. The libraries contained small fragmentsand ATAC-seq banding pattern products. **Figure S3**. Library quality assessment. Transcription start siteenrichment scores and unique fragment numbers were plotted in Aand Cby the reference genomes. Colored nuclei with doublet enrichment scores are illustrated in Band D. Panel E shows insert fragment length distributions. **Figure S4**. Nuclear fragment alignment status.The aligned read numbers to human or mouse references per nucleus were plotted, andthe aligned read number per nucleus was colored by NuHash count status.** Figure S5**. Distributions over different genomic features of the peaks categorized by the number of detected cell clusters. Peak annotations for each peak category are illustrated. Only the Cnum_4 group showed clear enrichment in promoter/enhancer regions. **Figure S6**. Differences in peak characteristics by the number of clusters detected.Expression statusof the genes located near the peaks was plotted. The white rectangles and their bars indicate the mean expression plus or minus a standard deviation.The proportion of peaks that overlapped with TAPEs was investigated, along withcomparisons of hematopoietic transcription factor binding motif enrichment of Cnum_4 peaks compared to all bulk ATAC-seq peaks.Transcription factor binding motif enrichment between low- and high-intensity Cnum_4 peaks was compared.The percentage of peaks that overlapped with CTCF ChIP-seq peaksand the distribution of their absolute distances from the TSS of Cnum_4 peaks were plotted.The percent of peaks that overlapped with CTCF ChIP-seq peaks and PIRs is summarized.

**Additional file 3**. NuHash demultiplexing script.

**Additional file 4**. A list of identified TAPEs.

## Data Availability

The sequencing data (bulk ATAC-seq and snATAC-seq) have been submitted to the NCBI Gene Expression Omnibus (GEO; https://www.ncbi.nlm.nih.gov/geo) under accession number GSE227522 (bulk ATAC-seq) and Short Read Archive (SRA, https://www.ncbi.nlm.nih.gov/sra) under accession number PRJNA942954 (snATAC-seq). The detailed method of library preparation and the Perl script for demultiplexing NuHash used in this study are provided in the Additional information.

## Abbreviations

NuHash: Nucleus hashing
ATAC-seq: Assay for Transposase-Accessible Chromatin with high-throughput sequencing
ChIP-seq: Chromatin immunoprecipitation followed by next-generation sequencing
snATAC-seq: Single-nuclear ATAC-seq
scRNA-seq: Single-cell RNA-seq
UMI: Unique molecular identifier sequences
GEM: Gel beads-in-emulsion
TSS: Transcription start site
OCRs: Open-chromatin region
TADs: Topologically associating domains
PIRs: Promoter-interacting regions
FBS: Fetal bovine serum
PBS: Phosphate-buffered saline
TAPE: Transcriptionally active putative enhancers
pCHiC: Promoter capture Hi-C
