# Supplementary material for: An optimized approach for multiplexing single-nuclear ATAC-seq using oligonucleotide-conjugated antibodies
Source: Epigenetics Chromatin. 2023 Apr 28;16:14. doi: 10.1186/s13072-023-00486-7 (PMC10142415; doi:10.1186/s13072-023-00486-7)
Supplement: Supplementary file 2 — Additional file 2: Figure S1. Optimizing NuHash antibody concentration to the number of nuclei. We stained the human CD4 + T-cell nuclei with different concentrations of NuHash antibody and generated bulk ATAC-seqlibraries to assess the proportions of NuHash and ATAC-seq products.We stained with an antibodyat three different concentrations. The panels show the fragment distributions of each library before removing large fragments by size selection.We stained with two different antibodiesat a 0.01 µg/50 k nuclei concentration. The panel shows the fragment distribution after removing large fragments by size selection. Figure S2. Library fragment distributions of NuHash scATAC-seq. NuHash antibodies were used to generate scATAC-seq libraries by multiplexing twoand four samples. The panels show the fragment length distributions of the scATAC-seq library final products. The libraries contained small fragmentsand ATAC-seq banding pattern products. Figure S3. Library quality assessment. Transcription start siteenrichment scores and unique fragment numbers were plotted in Aand Cby the reference genomes. Colored nuclei with doublet enrichment scores are illustrated in Band D. Panel E shows insert fragment length distributions. Figure S4. Nuclear fragment alignment status.The aligned read numbers to human or mouse references per nucleus were plotted, andthe aligned read number per nucleus was colored by NuHash count status. Figure S5. Distributions over different genomic features of the peaks categorized by the number of detected cell clusters. Peak annotations for each peak category are illustrated. Only the Cnum_4 group showed clear enrichment in promoter/enhancer regions. Figure S6. Differences in peak characteristics by the number of clusters detected.Expression statusof the genes located near the peaks was plotted. The white rectangles and their bars indicate the mean expression plus or minus a standard deviation.The proportion of peaks that overlapped with TAPEs was inv [file 13072_2023_486_MOESM2_ESM.docx]

**Supplementary Figures**

**Supplementary Figure 1**


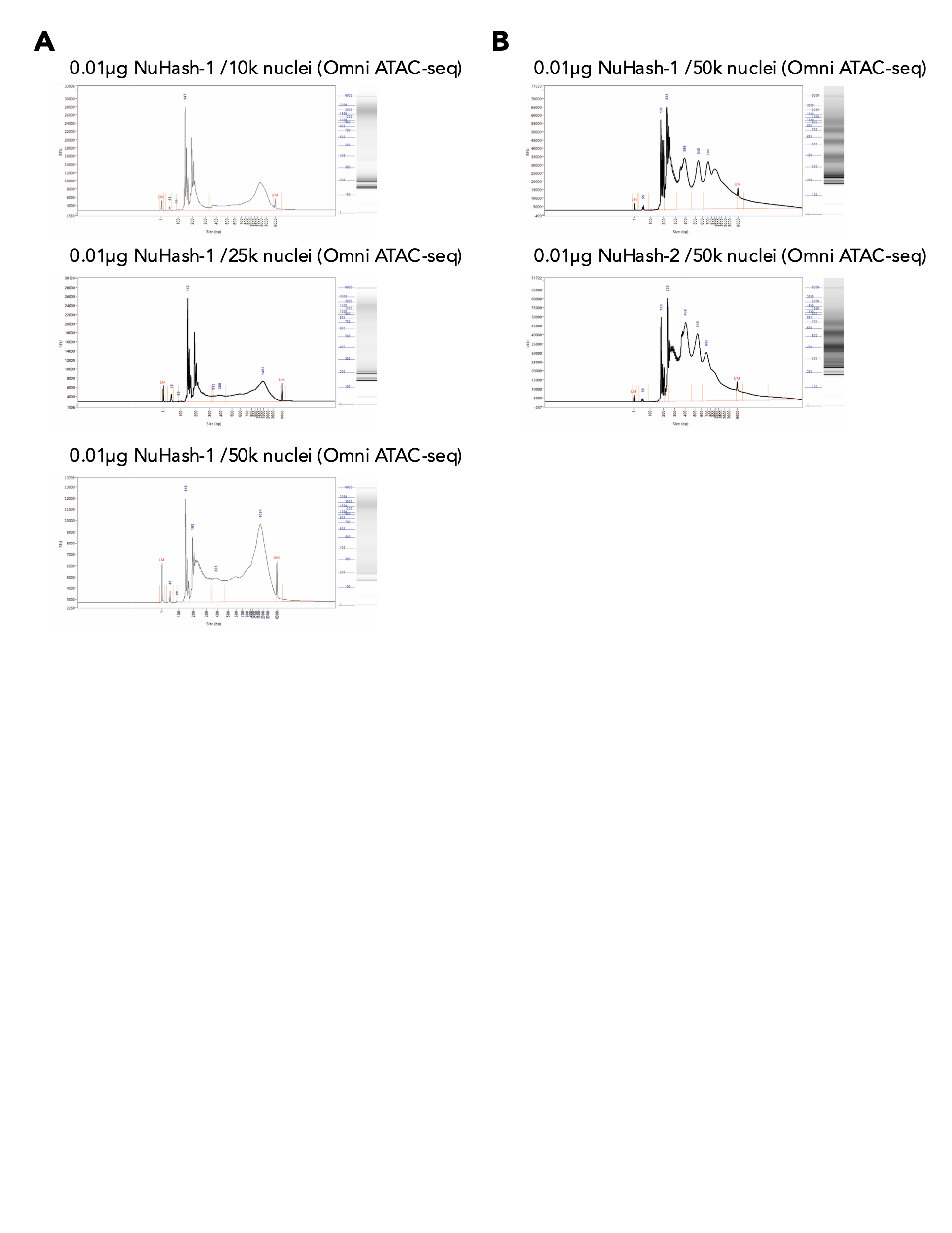


**Supplementary Figure 1: Optimizing NuHash antibody concentration to the number of nuclei.**

We stained the human CD4+ T-cell nuclei with different concentrations of NuHash antibody and generated bulk ATAC-seq (Omni- ATAC-seq) libraries to assess the proportions of NuHash and ATAC-seq products. **(A)** We stained with an antibody (NuHash-1) at three different concentrations. The panels show the fragment distributions of each library before removing large fragments by size selection. **(B)** We stained with two different antibodies (NuHash-1 and NuHash-2) at a 0.01 µg/50k nuclei concentration. The panel shows the fragment distribution after removing large fragments by size selection.

**Supplementary Figure 2**

**
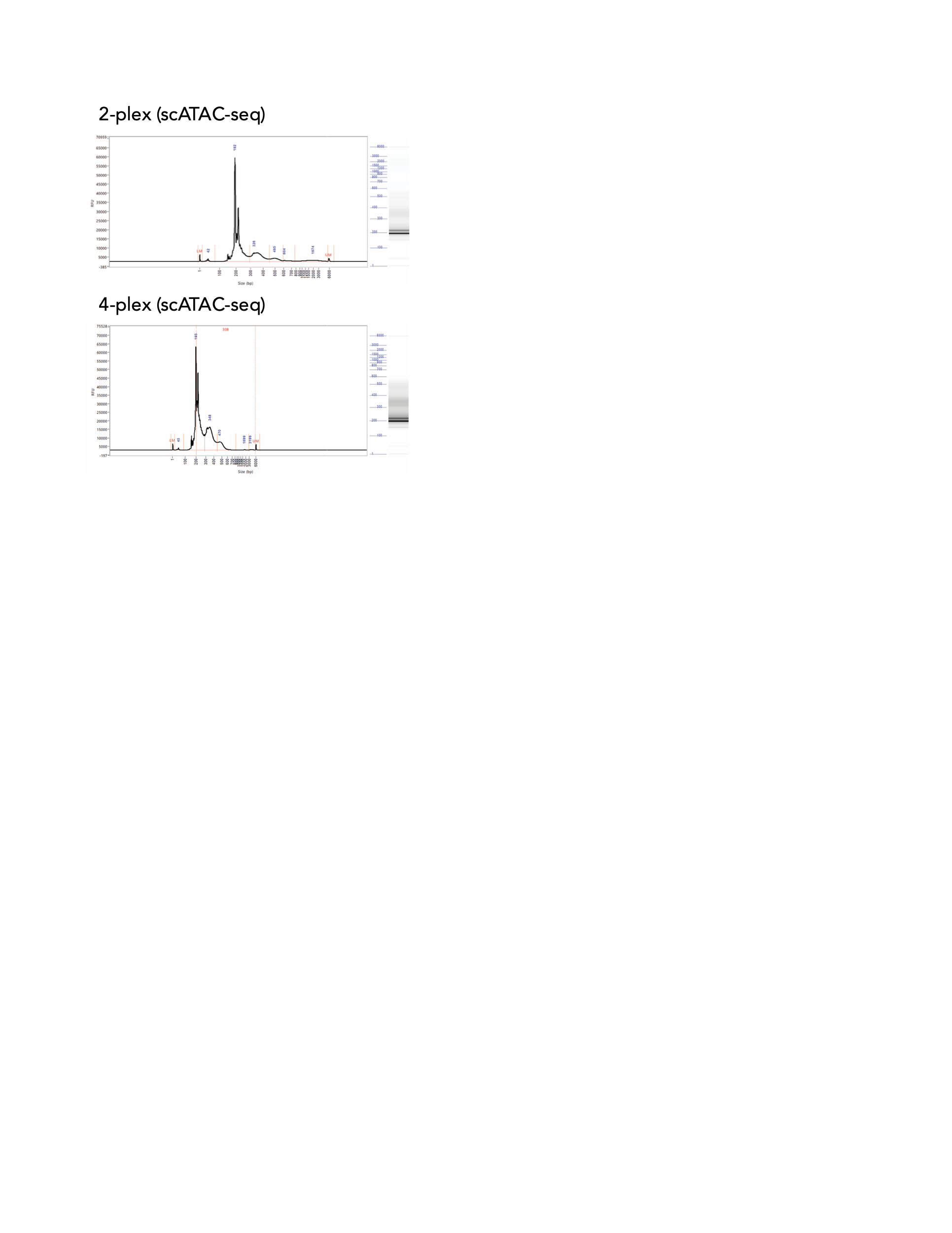
**

**Supplementary Figure 2: Library fragment distributions of NuHash scATAC-seq.**

NuHash antibodies were used to generate scATAC-seq libraries by multiplexing two (human and mouse samples, 2-plex) and four samples (two each human and mouse samples, 4-plex). The panels show the fragment length distributions of the scATAC-seq library final products. The libraries contained small fragments (NuHash products) and ATAC-seq banding pattern products.

**Supplementary Figure 3**

**
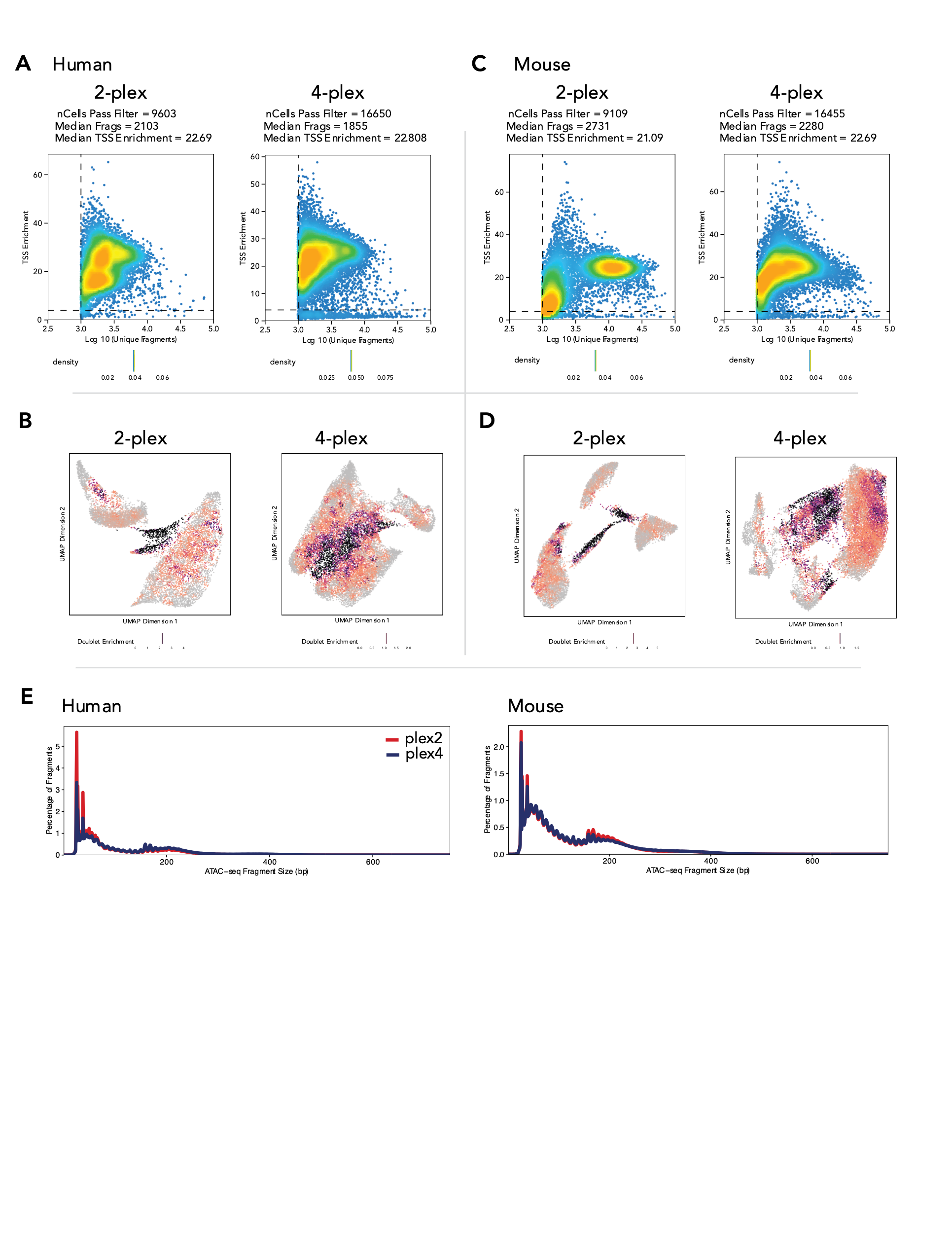
**

**Supplementary Figure 3: Library quality assessment.**

Transcription start site (TSS) enrichment scores and unique fragment numbers were plotted in **A** (human) and **C** (mouse) by the reference genomes. Colored nuclei with doublet enrichment scores are illustrated in **B**(human) and **D**(mouse). Panel **E** shows insert fragment length distributions.

**Supplementary Figure 4**

**
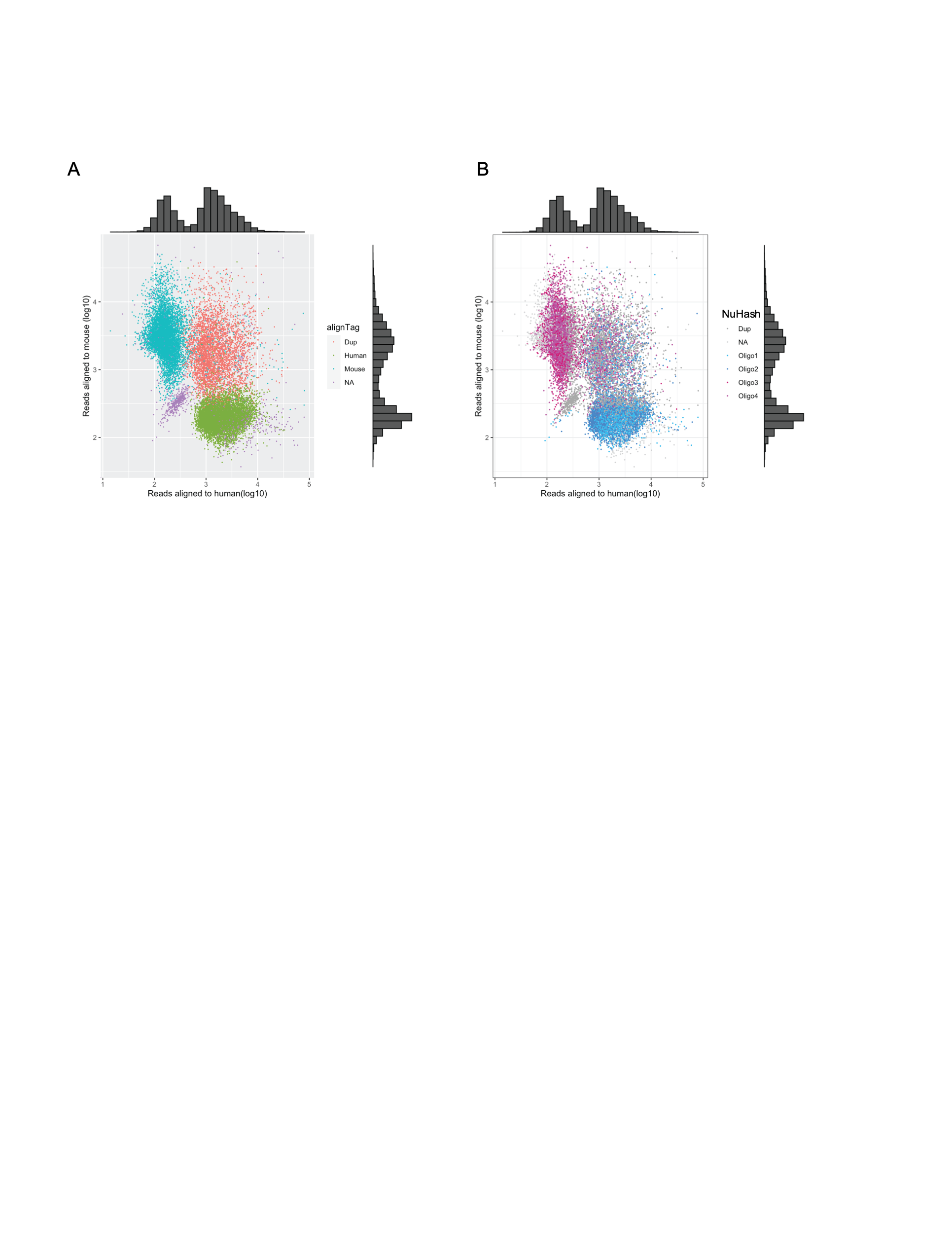
**

**Supplementary Figure 4: Nuclear fragment alignment status (4-plex).**

(**A**) The aligned read numbers to human or mouse references per nucleus were plotted, and (**B**) the aligned read number per nucleus was colored by NuHash count status.

**Supplementary Figure 5**

**
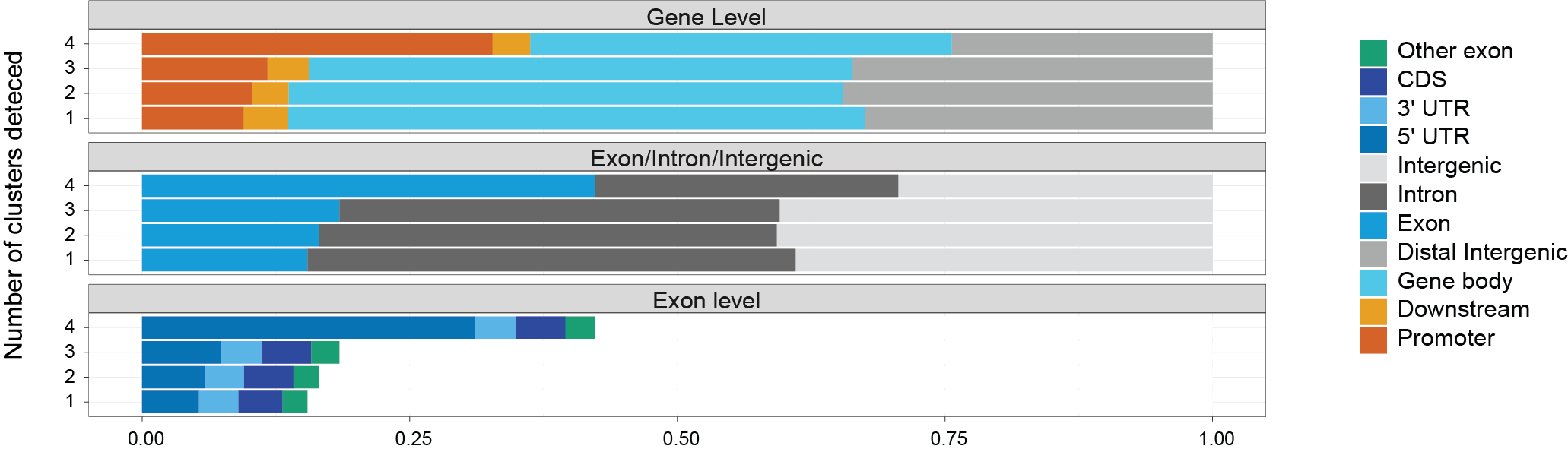
**

**Supplementary Figure 5: Distributions over different genomic features of the peaks categorized by the number of detected cell clusters.**

Peak annotations for each peak category are illustrated. Only the Cnum_4 group showed clear enrichment in promoter/enhancer regions.

**Supplementary Figure 6**

**
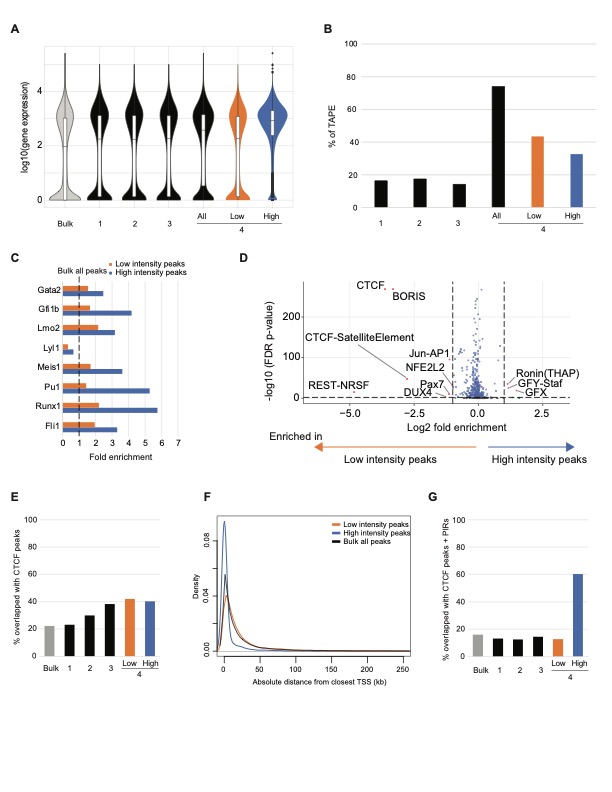
**

**Supplementary Figure 6: Differences in peak characteristics by the number of clusters detected.**

**(A)** Expression status (log_10_) of the genes located near the peaks was plotted. The white rectangles and their bars indicate the mean expression plus or minus a standard deviation. **(B)** The proportion of peaks that overlapped with TAPEs was investigated, along with **(C)** comparisons of hematopoietic transcription factor binding motif enrichment of Cnum_4 peaks compared to all bulk ATAC-seq peaks. **(D)** Transcription factor binding motif enrichment between low- and high-intensity Cnum_4 peaks was compared. **(E)** The percentage of peaks that overlapped with CTCF ChIP-seq peaks **(F)** and the distribution of their absolute distances from the TSS of Cnum_4 peaks were plotted. **(G)** The percent of peaks that overlapped with CTCF ChIP-seq peaks and PIRs is summarized.
